# Predicted Skeletal Muscle Mass and 4-Year Cardiovascular Disease Incidence in Middle-Aged and Elderly Participants of IKARIA Prospective Epidemiological Study: The Mediating Effect of Sex and Cardiometabolic Factors

**DOI:** 10.3390/nu12113293

**Published:** 2020-10-27

**Authors:** Christina Chrysohoou, Matina Kouvari, George Lazaros, John Varlas, Kyriakos Dimitriadis, Marina Zaromytidou, Constantina Masoura, John Skoumas, Manolis Kambaxis, Nikos Galiatsatos, Aggelos Papanikolaou, Panagiotis Xydis, Konstantinos Konstantinou, Christos Pitsavos, Konstantinos Tsioufis, Christodoulos Stefanadis

**Affiliations:** 1 Cardiology Clinic, Hippokration Hospital, Medical School, Kapodistrian University of Athens, 11527 Athens, Greece; matinakouvari4@gmail.com (M.K.); glaz35@hotmail.gr (G.L.); dimitriadiskyr@yahoo.gr (K.D.); mzaromit@yahoo.gr (M.Z.); kmasoura@gmail.com (C.M.); skoumasj@yahoo.gr (J.S.); info@medicalcdiet.gr (M.K.); nikosgaliatsatos1957@gmail.com (N.G.); agepap25@hotmail.com (A.P.); panosxydis@yahoo.gr (P.X.); kostiskon@gmail.com (K.K.); cpitsavo@med.uoa.gr (C.P.); ktsioufis@gmail.com (K.T.); stefanadischristodoulos@gmail.com (C.S.); 2 Department of Nutrition and Dietetics, Harokopio University of Athens, 17676 Athens, Greece; 3 Immunology, Scientific Support Department, MEDICON Hellas S.A., Gerakas, 15344 Attica, Greece; cvarlas@gmai.com

**Keywords:** aging, women, heart disease, gender, lean mass, body composition, obesity, primary prevention

## Abstract

The sex-specific effect of skeletal muscle mass (SMM) index (SMI) on 4-year first fatal/non-fatal cardiovascular disease (CVD) event in free-of-disease individuals was examined. In 2009, *n* = 1411 inhabitants (mean age = 64(12)) from Ikaria were selected. Follow-up was performed in 2013. SMI was created to reflect SMM through appendicular skeletal muscle mass (indirectly calculated through formulas) divided by body mass index (BMI). Fifteen and six tenths percent of participants exhibited CVD (19.8% in men/12% in women, *p =* 0.002). Significant U-shape trends were observed in participants >65 years old and women irrespective to age confirmed through multi-adjusted Cox regression analysis; in age >65 years, Hazard Ratio (HR)_(2nd vs. 1st SMI tertile)_ = 0.80, 95% Confidence Interval (95%CI) (0.45, 0.96) and in women HR_(2nd vs. 1st SMI tertile)_ = 0.71, 95% CI (0.33, 0.95), while, as for the 3rd SMI tertile, no significant trends were observed. Mediation analysis revealed that mediators of the aforementioned associations in men were the arterial distensibility and total testosterone, while, in women, inflammation, insulin resistance, and arterial distensibility. High SMM accompanied by obesity may not guarantee lower CVD risk. Specific cardiometabolic factors seem to explain this need for balance between lean and fat mass.

## 1. Introduction

Aging is accompanied by a series of morphological and functional alterations which take place slowly over time. In humans, the aging process is altered or accelerated when metabolic and cardiovascular disease (CVD) occur. Skeletal muscle mass (SMM) alterations are among these physiological changes with decline normally starting from middle age or earlier and progressing in more advanced age [1]; in particular, SMM declines with a rate of >3% per decade starting from the age of 30 years old. 

Anthropometric parameters are highly discussed in relation to chronic diseases, yet mostly in terms of abnormal weight status or adiposity; suggesting from a lineal trend, in younger, free-of-disease individuals, to a U-shape trend in older adults with or without established diseases [2]. Focusing on the later paradoxical association, the role of fat-free mass is highly discussed yet with inadequate evidence [3,4]. In this respect, SMM accounts almost the half of body mass possessing an active role in various metabolic pathways [5]. Hence, hypotheses exist regarding the potential association of SMM with lower inflammation [6], better insulin sensitivity [7], lower arterial stiffness [8], and hormone status (principally in terms of testosterone levels) [9]. However, limited evidence exists regarding the direct effect of SMM on major events, as well as the potential mediating effect of the aforementioned paths. This literature gap inherently deters robust conclusions, even more when it comes to sex-related specifications [10].

The aim of the present work was to evaluate the association between skeletal muscle mass index (SMI)—a body mass index (BMI)-adjusted index of SMM (the higher SMI values, the higher SMM) with 4-year first fatal/non-fatal CVD event in apparently healthy men and women of middle-aged to advanced age, using a sample from prospective epidemiological Ikaria study. Within the last decade, Ikaria island inhabitants have been recognized by the “Blue Zones” as having among the highest longevity rates universally, with high percentage of healthy aging [11]. To the best of our knowledge, this is one of the very few works with the potential to provide evidence on the role of SMM on the CVD risk of middle-aged and mostly elderly free-of-disease individuals. We posed three a priori research hypotheses: a. CVD incidence will be progressively reduced passing from lower to higher SMI values; b. the examined association will be different between sexes due to physiological differences in body-composition metrics; and **c.** cardiometabolic paths related with inflammation, insulin resistance, hormone status and arterial stiffness are involved in the examined association.

## 2. Materials and Methods 

### 2.1. Study Sample

Ikaria study is a prospective, observational cohort investigation initiated in 2009. At baseline (June 2009–October 2009), *n* = 1411 middle-aged and elderly volunteers residing in the Ikaria island agreed to participate (84% participation rate). Of the enrolled participants, *n* = 668 (47%) were men (65 ± 13 years), and *n* = 743 (53%) were women (65 ± 14 years). For our primary analysis, we excluded *n* = 182 participants with positive baseline CVD history and *n* = 87 without follow-up metrics on CVD for a final study sample of *n* = 1142 (*n* = 527 women).

### 2.2. Bioethics

The study was approved by the Medical Research Ethics Committee of 1st Cardiology Clinic of University of Athens at Hippokration General Hospital and was carried out in accordance with the Declaration of Helsinki (1989) of the World Medical Association (Scientific Committee and Directory Board of Hippokration Hospital decision 10/29-06-2009). All participants were informed about the aims of the study, agreed to participate, and signed an informed consent.

### 2.3. SMI Calculation

SMM was calculated through appendicular skeletal muscle mass (ASM) based on the equation by Lee and colleagues; ASM = (0.244*weight) + (7.8*height) + (6.6*sex) − (0.098*age) + (race − 3.3) (sex: Women = 0/men = 1; race: White/Hispanic = 0/black = 1.9/Asian = −1.6) [12]. This indicator was divided by BMI to create SMI. SMI tertiles were created specified for men and women with 1st tertile corresponding to the lowest muscle mass.

### 2.4. Other Baseline Measurements

The biochemical evaluation was carried out in a laboratory that followed the criteria of the World Health Organization Reference Laboratories. The homeostasis model assessment (HOMA) for the description of glucose regulation was accomplished by the use of the equation written below: HOMA-IR = (glucose x insulin)/405 glucose in mg/dl, insulin in μU/mL. Total serum testosterone was measured by a solid-phase, competitive chemiluminescent enzyme immunoassay method, IMMULITE 2000. Aortic distensibility was calculated from the aortic diameters and aortic pressure or brachial artery pressure using the formula [2 × (change in aortic diameter)/(diastolic aortic diameter) × (change in aortic pressure)] [13].

### 2.5. Endpoint and Follow-up Evaluation

During June-July 2013, 4-year follow-up was performed. Information from all individuals was retrieved. The studied endpoint was 4-year first fatal/non-fatal CVD event. CVD event was defined as development of coronary heart disease (myocardial infarction, angina pectoris, other identified forms of ischemia -WHO-ICD coding 410–414.9, 427.2, 427.6-) and chronic arrhythmias -WHO-ICD coding 400.0–404.9, 427.0 −427.5, 427.9-), and development of stroke (WHO-ICD coding 430–438).

### 2.6. Statistical Analysis

Categorical variables are presented as absolute (n) and relative frequencies (%). Continuous variables are presented as mean ± standard deviation. Associations between normally distributed variables and SMI tertiles were evaluated through one-way analysis of variance. Whether these variables were normally distributed was tested through P-P plot and equality of variances through Levene’s test. For non-normally distributed variables, Kruskal–Wallis test was used. Associations between categorical variables and SMI tertiles were tested with chi-squared test. HRs and their corresponding 95%CIs for 4-year CVD event were evaluated through Cox-regression analysis. Proportional hazards’ assumption was graphically tested. Kaplan-Meier survival curves, illustrating 4-year CVD event across SMI tertiles, were constructed and compared using the long-rank test. Multi-adjusted linear regression models were applied to test the association between SMI and various continuous variables (per 1 unit). STATA software, version 14 (MP & Associates, Sparta, Greece) was used for all statistical analyses. We deemed statistical significance at *p-* value *< 0.05*.

## 3. Results

Overall 4-year CVD event in men and women participants of IKARIA study (*n* = 1147) was 15.6% (*n* = 178) (19.8% in men and 12% in women, *p =* 0.002).

Table 1 depicts participants’ baseline characteristics and 4-year CVD incidence according to SMI tertiles. Ranking from 1st to 3rd SMI tertiles higher waist circumference and waist-to-hip ratio metrics were observed (all ps < 0.001)—probably driven by the increased BMI. Participants assigned in 2nd SMI tertile, even with overweight status, had the best –on the whole– inflammation status, insulin resistance, liver function and arterial distensibility profile. Total testosterone levels were higher in participants assigned in the 3rd SMI tertile (*p =* 0.05).Participants assigned in 3rd SMI tertile had the lowest likelihood to develop CVD compared with their 1st SMI counterparts (*p =* 0.04), yet being quite close to participants assigned in 2nd SMI tertile. When only participants >65 years old were taken into account, those with moderate SMI (2nd tertile) had the lowest CVD rates (*p =* 0.01). In both cases, women assigned principally in the 2nd SMI tertile had about 40% lower likelihood to suffer from a cardiac episode compared with a man assigned in the same tertile (all ps < 0.05).

Nested Cox regression models to evaluate the association of SMI status and 4-year first CVD event are presented in Table 2. In crude model, participants at the 2nd and 3rd tertile presented almost 24% and 30% lower risk for developing CVD within follow-up period compared with their counterparts in 1st tertile (*p* < 0.001).This association, even slightly weaker, was retained after adjusting for various clinical, lifestyle, and sociodemographic factors (Models 2–5, all ps < 0.05). Separate adjustments for inflammatory markers, insulin resistance, arterial distensibility, and total testosterone levels revealed that the aforementioned trends were retained, yet they did not reach the level of significance (all ps > 0.05) (Model 6–9). Models were additionally rerun setting participants at the 2nd SMI tertile as a reference group and examined it against participants assigned in the 3rd tertile; multi-adjusted analysis (on the adjustment basis of Model 5) revealed no significant differences between the two groups [HR, 95% CI: 1.19 (0.85, 2.26), *p =* 0.15] (data not shown). 

In the formal analysis of interaction, a significant heterogeneity on the basis of sex and age was produced (all ps for interaction < 0.05) as regards the association of SMI with 4-year first CVD event accompanied by a triple interaction between age, sex, and SMI, as well (*p* for interaction = 0.004). Hence, sensitivity analysis was performed and results are presented in Table 3. In particular, when the multi-adjusted models used in Table 2 were rerun excluding participants <65 years old, those assigned in the 2nd SMI tertile had about 20% lower risk to develop CVD within the 4-year period compared with their 1st tertile counterparts (*p* = 0.01). This trend was lost when adjusting for inflammatory markers, insulin resistance, arterial distensibility, and total testosterone. Stratification by sex revealed that, while in men 3rd SMI tertile was independently associated with lower CVD risk compared with 1st tertile in case of women, a U-shape trend was suggested with participants assigned in 2nd tertile having about 29% lower risk to develop CVD (*p* for non-linear/U-shape trend = 0.04). Moreover, moderators in men were the arterial distensibility and total testosterone, while, in women, inflammation, insulin resistance, and arterial distensibility. Finally, focusing on men and women >65 years old, a significant U-shape trend of SMI was retained only in women; the association was alleviated when adjusting for inflammation, arterial distensibility, and total testosterone. In men, no significant trends, either linear or non-linear, were revealed.

The multi-adjusted associations between various surrogate markers and SMI are summarized in Table 4. In the context of inflammation biomarkers, significant inverse associations were observed for both men and women in terms of ultra-sensitive C-reactive protein (usCRP) and white blood cells (WBC) (all ps < 0.05). As regards HOMA-IR, this was inversely associated with SMI in the total sample, as well as in women. Total testosterone and arterial distensibility were also correlated with SMI yet only in case of total sample and men. When only the subgroup of participants >65 years old was taken into account, the aforementioned inflammatory markers were significantly related with SMI principally in women, while arterial distensibility and total testosterone were significantly correlated with both men’s and women’s SMI.

**Figure 1:**
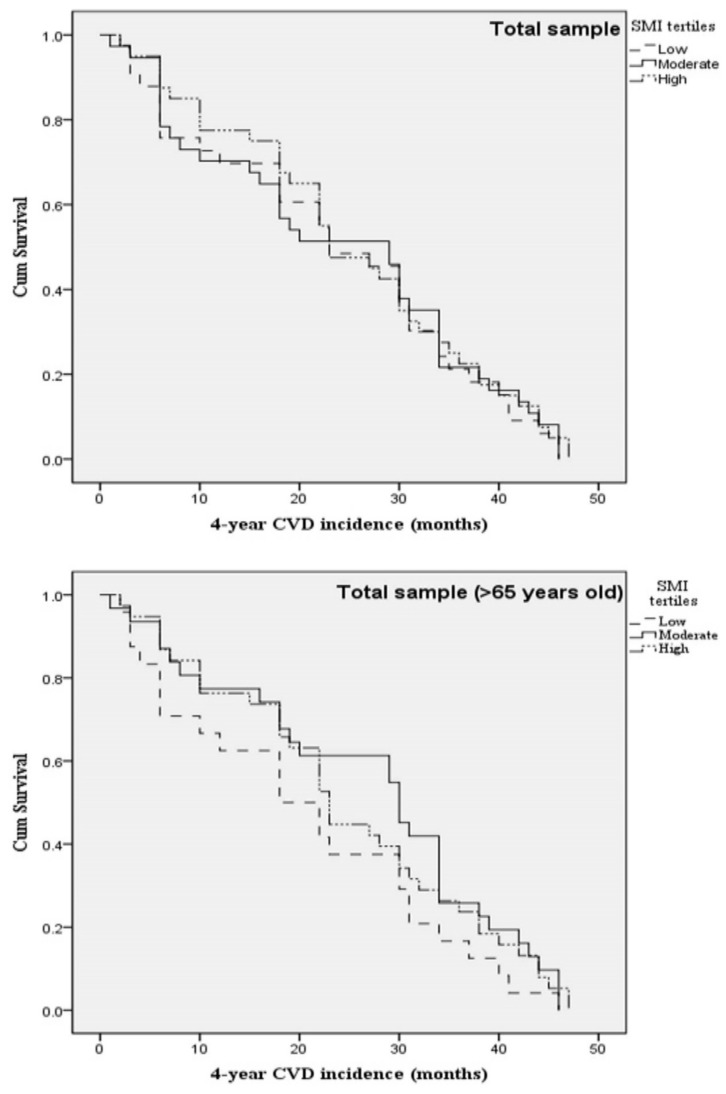
Kaplan-Meier survival curves for 4-year first fatal/non-fatal CVD event in apparently healthy men and women according to SMI tertiles and age status. Models were adjusted for (age), sex, current smoking, physical activity, diabetes mellitus, dyslipidemia, hypertension, and family history of CVD. Abbreviations: Cardiovascular disease (CVD); Skeletal muscle mass index (SMI).

**Figure 2:**
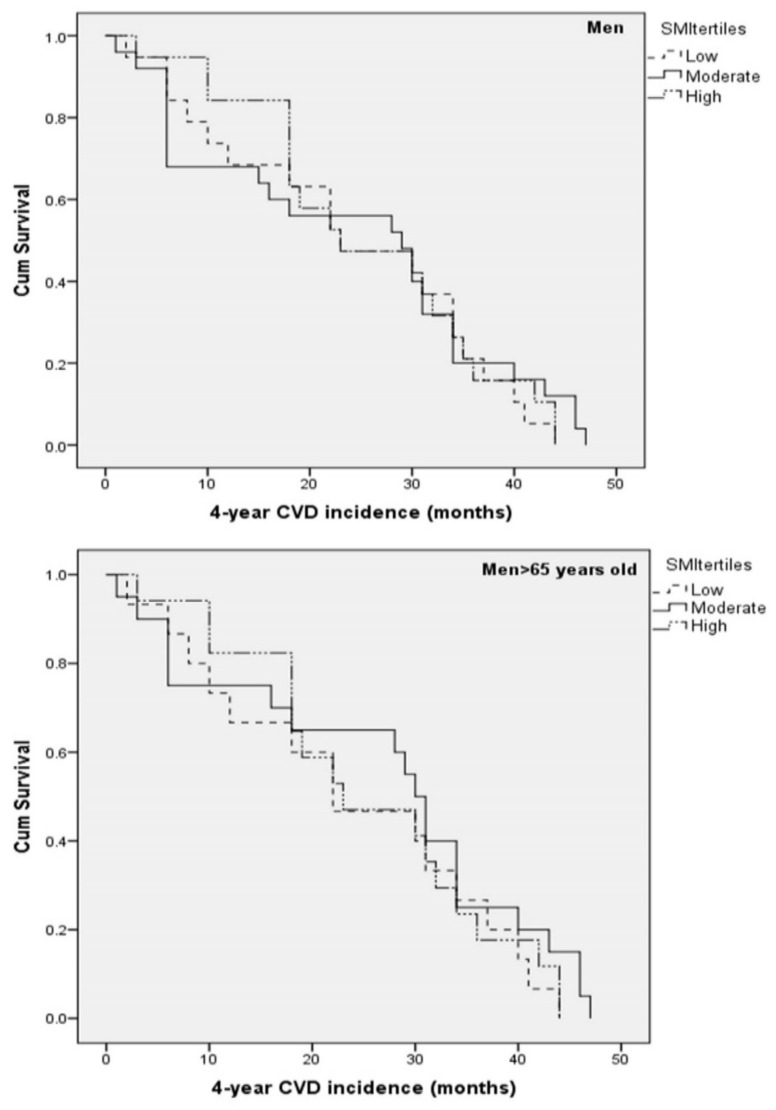
Kaplan-Meier survival curves for 4-year first fatal/non-fatal CVD event in apparently healthy men according to SMI tertiles and age status. Models were adjusted for (age), current smoking, physical activity, diabetes mellitus, dyslipidemia, hypertension, and family history of CVD. Abbreviations: Cardiovascular disease (CVD); Skeletal muscle mass index (SMI).

**Figure 3:**
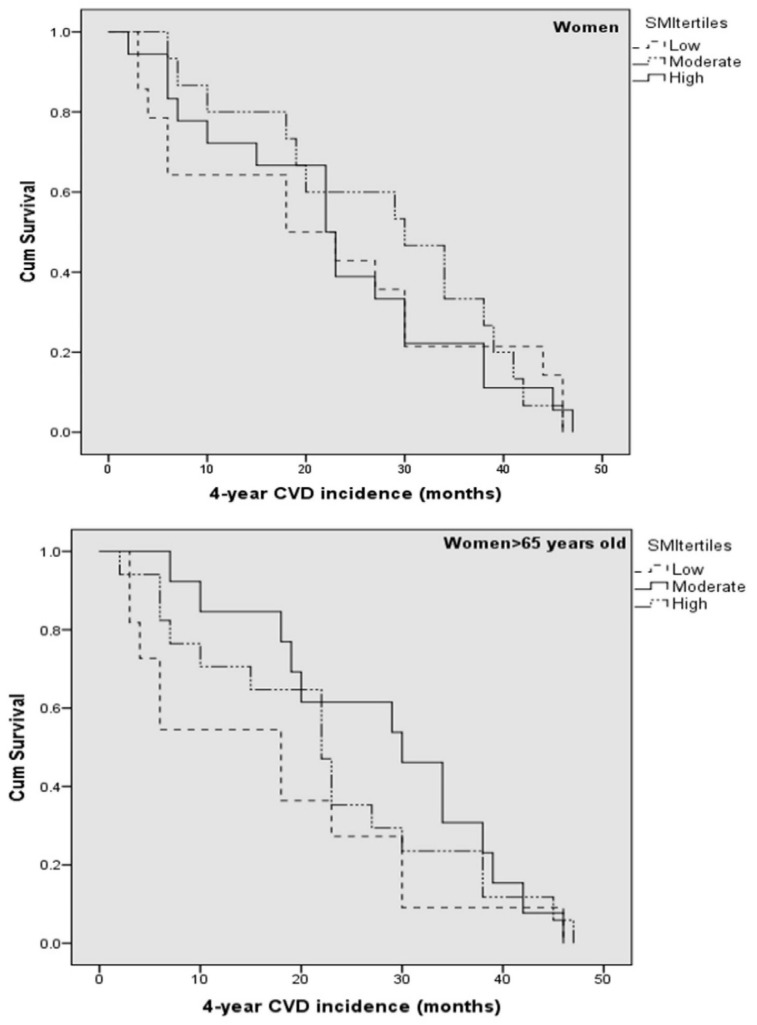
Kaplan-Meier survival curves for 4-year first fatal/non-fatal CVD event in apparently healthy women according to SMI tertiles and age status. Models were adjusted for (age), current smoking, physical activity, diabetes mellitus, dyslipidemia, hypertension, and family history of CVD. Abbreviations: Cardiovascular disease (CVD); Skeletal muscle mass index (SMI).

Kaplan-Meier survival curves were constructed to evaluate the 4-year first CVD event rate in the total sample, as well as separately for men and women (including or excluding participants <65 years old), according to SMI status. Survival curves are illustrated in Figure 1 for total sample, in Figure 2 for men, and in Figure 3 for women.

## 4. Discussion

Partially in contrast with our initial hypothesis, the findings revealed here, in a sample of middle aged and elderly participants, suggest a *U*-shape trend between SMI and CVD onset within a 4-year period, principally in women (irrespective to age), as well as individuals over 65 years old. Notably, the linear trend between SMI and CVD that we initially hypothesized was observed only in men, mainly the younger ones. In addition, this work stands among the very few that provide evidence on the moderating effect of various cardiometabolic factors, in addition to the highly discussed inflammation and insulin resistance, with specifications according to participants’ age and sex, suggesting testosterone and arterial dispensability as potential underlying paths that explain the effect of SMM on primary CVD prevention. 

Much as elevated BMI presents deleterious effects on CVD onset, weight status in advanced age raises mixed messages and paradoxical associations [14,15]. Notably, in the present work, even if participants assigned in the 2nd and 3rd tertile had the lowest CVD risk, a linear trend could not be supported since no significant differences were observed between 2nd and 3rd SMI tertile. Additionally, it was of interest that when participants <65 years were excluded from our primary analysis, a U-shape trend was observed. Our findings come in line with recent works [3,16,17,18]. These works imply that high muscle mass accompanied by obese and/or excess-body-fat status may not be that protective. What should be outlined here is that these outcomes were generated from samples not only with advanced age but also with established CVD, contributing to an increased catabolic rate, probably higher than in the study sample used here.

This work is one of the very first that investigated sex differences in the association between muscle mass and CVD in individuals mostly at advanced age. Sensitivity analysis revealed that women in 2nd SMI tertile presented the lowest risk to develop CVD principally in case of age > 65 years old. A sex-based analysis from ATTICA study revealed that in women adiposity is more important CVD predictor than lean mass [17]; this partially comes in line with findings raised here where increased adiposity seemed to alleviate the protective role of SMM. Similar analyses from the GREECS and Hellenic Heart Failure study, even in individuals with established CVD, agreed on the potential existence of a U-shape trend [16,17]. According to recent evidence, women seem to be more susceptible to age-dependent loss of muscle mass, grid, and function in terms of sarcopenia [19,20], probably related with biological and lifestyle variances [10]. An additional finding here was that mostly overweight women with moderate SMI presented the lowest CVD risk. Meanwhile, the obtained knowledge is that BMI-related paradoxical association is more evident in women where a non-linear trend has been suggested in advanced age or established chronic diseases [21,22]. The added value of the present work is related with highlights that women’s “overweight paradox” in advanced age may be attributed not only to their resistance to adiposity but also to sex-specific responses of muscle mass.

The mechanisms through which SMM exerts its potential cardioprotective effects has been inadequately investigated. Our work adds to the hitherto literature suggesting potential mediators. Firstly, it was of interest that participants assigned in 2nd SMI tertile had the best inflammation-related profile. This observation comes in line with previous works [16,23,24], further enhancing the generation of a biologically plausible hypothesis regarding the impact of systemic inflammation upon the deterioration of muscle mass and function. Secondly, insulin resistance was to mediate the protective role of SMI on cardiac health principally in women, probably the younger ones. Muscle mass is an important determinant of glucose and energy homeostasis determined by the balance between protein synthesis and breakdown in the tissue. However, resistance to the anabolic action of insulin has been demonstrated even in older individuals of normal muscle mass and may, therefore, precede the physical manifestations of sarcopenia [7].

In this work, aortic distensibility was related to SMI in both sexes revealing the common pathways between arterial aging and muscle mass preservation. Additionally, overweight participants with moderate SMI had the highest arterial distensibility. This may be another underlying mechanism through which the suggested U-shape trend is exerted. The independently inverse association between arterial stiffness and SMM, in contrast with the positive association with adiposity has been previously reported [25]. It is well known that aging causes progressive stiffness, dilatation, and lengthening of the arteries; thus, low arterial distensibility has long been recognized as an indicator of atherosclerosis and future cardiovascular events [26]. Furthermore, in participants in IKARIA study, aortic stiffness increases gradually with age; however, in people above the age 50 years old, aortic stiffening seems to be decelerated as it is more preserved than reference values [26]. This finding may be attributed to either a favorable hemodynamic pattern or to beneficial genetic and metabolic backgrounds that are implicated in the process of both longevity and aortic stiffness, and this also reflected in preserved muscle mass [26].

Aging is related with a significant reduction in sex hormones. Androgen deficiency, along with lack of exercise and poor nutrition, may be among the modifiable contributors to sarcopenia [27]. Indeed, low testosterone levels are associated with unfavorable body composition changes. On the other side, epidemiological studies show that low testosterone levels are associated with atherosclerosis, principally in men [27]. Considering that increased muscle mass is mostly associated with increased testosterone levels, this linkage could mediate the effect on cardiac health. The analysis performed here revealed that total testosterone generally alleviated the independent protective role of SMI on CVD. Moreover, there is a different nature of aging between sexes (endocrine, testosterone) that also affects its interrelation to the metabolic-CVD pathway. Here, from a sex-based approach, total testosterone may justify the reason for which the suggested U-shape trend was not the case in men, considering that the 3rd SMI tertile had the highest testosterone levels. In women, mixed outcomes were generated. Specifically, testosterone seemed to mediate the examined association only in advanced age, taking into account that, in women, hyperandrogenism is related with increased insulin resistance, metabolic syndrome, and CVD, which may explain the observed U-shape trend here, as well as in previous works [28]. Hyperandrogenism is usually observed in obese women with diabetes mellitus and polycystic ovarian syndrome; in the present work, participants in the 3rd SMI tertile had the highest rates of diabetes and metabolic syndrome, which enhances this hypothesis.

### Limitations and Strengths

The main strength here that compensates the following limitations is that this in one of the very few prospective studies that evaluated the sex-specific effect of SMI on 4-year first fatal/non-fatal CVD incidence in apparently healthy men and women of advanced age. However, several limitations should be presented. Specifically, only baseline measurements were taken into account for our research hypothesis. Moreover, imaging data or skinfold metrics were not available, and SMM calculation was based on population equations that may under- or over-estimate the actual body composition rates; nonetheless, these formulas have been validated and present good agreement with the classical dual-energy X-ray absorptiometry method [12].

## 5. Conclusions

The management of individuals at advanced age with increased weight remains a challenging scientific field. Our work sought to address this issue through the provision of specific key findings. Firstly, although the deterioration of a healthy muscle mass may be an independent protective factor against CVD, in the case of obesity, and probably excess adiposity, increased SMM does not seem to guarantee better health status. Secondly, various alternate and probably interchangeable metabolic mechanisms were revealed to mediate the examined association, probably justifying the need for a balance between lean and fat mass. Lastly, the sex-specific remarks revealed here highlight the need for specification of anthropometric features that better predict men’s and women’s cardiac health.

## Figures and Tables

**Table 1:** Baseline sociodemographic, clinical, anthropometric, biochemical and lifestyle characteristics of free of cardiovascular disease men and women from the IKARIA study according to estimated skeletal muscle mass index tertiles (*n* = 1141).

Baseline Characteristics	Skeletal Muscle Mass Index Tertiles			
	**1st Tertile**	**2nd Tertile**	**3rd Tertile**	***p*-Value**
*N*	371	374	396	
**Sociodemographic factors**				
Age, years	64 (12)	64 (13)	63 (14)	0.22
Male sex, %	46	46	47	0.97
**Anthropometric factors**				
Body mass index, kg/m^2^	23.9 (2.1)	28.0 (1.5)	33.6 (3.7)	<0.001
Waist circumference, cm	91 (11)	101 (9)	111 (11)	<0.001
Waist-to-hip ratio	0.91 (0.09)	0.95 (0.09)	0.97 (0.09)	<0.001
**Lifestyle factors**				
Physical inactivity, %	30	38	35	0.93
MedDietScore, range 0–55	34.4 (0.6)	34.5 (0.6)	34.3 (0.6)	0.04
Weekly alcohol consumption, %	61	62	63	0.81
Current smoking, %	23	30	38	<0.001
**Clinical factors**				
History of hypertension, %	30	44	53	<0.001
History of diabetes mellitus, %	22	17	85	<0.001
History of hypercholesterolemia, %	33	36	44	0.00
Metabolic syndrome, %	16	39	55	<0.001
Family CVD history, %	40	37	40	0.70
**Inflammation/coagulation markers**				
Ultra-sensitive C-Reactive Protein, mg/L	3.4 (3.9)	1.8 (2.1)	3.0 (3.2)	<0.001
Interleukin 6, pg/dL	3.8 (11.5)	4.4 (13.0)	4.7 (24.0)	0.87
Tumor necrosis factor-alpha, pg/mL	1.51 (0.50)	1.53 (0.50)	1.62 (0.49)	0.63
White blood cells, 10^3^ counts	6.4 (1.6)	5.9 (1.5)	6.2 (1.4)	<0.001
**Aortic stiffness markers**				
Aortic distensibility, 1000*mmHg^−1^	1.55 (1.47)	1.70 (1.07)	1.67 (1.46)	<0.001
Pulmonary pressure, mmHg	31 (6)	31 (6)	30 (6)	0.02
**Liver function markers**				
Alanine transaminase, U/L	14.8 (6.8)	15.9 (7.1)	18.3 (10.4)	<0.001
Aspartate transaminase, U/L	22.7 (7.9)	22.6 (11.4)	22.0 (6.4)	0.45
**Glucose/insulin homeostasis markers**				
Fasting glucose, mg/dL	96 (23)	104 (31)	107 (28)	<0.001
HOMA-IR	0.81 (0.67)	1.04 (1.44)	1.80 (3.04)	<0.001
**Lipid markers**				
Total cholesterol, mg/dL	205 (39)	205 (40)	204 (42)	0.97
High density lipoprotein cholesterol, mg/dL	45 (10)	46 (9)	50 (13)	<0.001
Triglycerides, mg/dL	127 (85)	147 (95)	160 (91)	<0.001
Low density lipoprotein cholesterol, mg/dL	128 (34)	129 (34)	129 (33)	0.89
**Hormones**				
Total testosterone, ng/dL	157 (19)	177 (20)	220 (242)	0.05
**4-year follow-up measurements**				
First fatal/non-fatal cardiovascular disease incidence	19.2	14.2	13.3	0.04
First fatal/non-fatal cardiovascular disease incidence (excluding participants <65 years old)	27.4	23.9	25.9	0.01
Woman-to-man cardiovascular disease incidence ratio	0.68	0.57	0.78	0.03
Woman-to-man cardiovascular disease incidence ratio (excluding participants <65 years old)	0.88	0.60	0.77	0.001


Data are presented as mean ± standard deviation (SD) or median (Interquartile Range) if normality was not met. *p*-values were obtained using one-way ANOVA for the normally distributed variables (age, body mass index), Kruskal–Wallis test for the rest quantitative variables, and chi-squared test for categorical variables. Abbreviations: Homeostatic Model Assessment of Insulin Resistance (HOMA-IR).

**Table 2:** Nested Cox-regression analysis models to evaluate the association of SMI with 4-year first fatal/non-fatal cardiovascular disease (CVD) event in apparently healthy men and women of IKARIA study (*n* = 1141).

Models	Model 1	Model 2	Model 3	Model 4	Model 5	Model 6	Model 7	Model 8	Model 9
	HR (95% CI)	HR (95% CI)	HR (95% CI)	HR (95% CI)	HR (95% CI)	HR (95% CI)	HR (95% CI)	HR (95% CI)	HR (95% CI)
SMI tertiles									
1st	Ref	ref	ref	ref	Ref	ref	ref	ref	ref
2nd	**0.76** **(0.43, 0.91**)	**0.82** **(0.52, 0.91)**	**0.85** **(0.52, 0.93**)	**0.86** **(0.40, 0.93)**	**0.87** **(0.45, 0.99**)	0.92 (0.52, 1.08)	1.28 (0.61, 2.65)	1.01 (0.52, 1.92)	1.09 (0.51, 2.32)
3rd	**0.70** **(0.36, 0.86)**	**0.74** **(0.43, 0.86)**	**0.75** **(0.45, 0.82)**	**0.78** **(0.48, 0.85**)	**0.83** **(0.37, 0.95)**	**0.89** **(0.41, 0.99)**	1.31 (0.55, 3.12)	0.83 (0.37, 1.88)	0.99 (0.39, 2.48)
Age, per 1 year	-	1.04 (1.02, 1.06)	1.04 (1.03, 1.06)	1.06 (1.03, 1.08)	1.04 (1.02, 1.06)	1.07 (1.00, 1.13)	1.06 (1.02, 1.10)	1.04 (1.02, 1.07)	1.05 (1.01, 1.10)
Male sex	-	1.74 (1.16, 2.59)	1.75 (1.15, 2.67)	1.76 (1.13, 2.74)	2.10 (1.22, 3.59)	1.99 (0.91, 4.33)	1.70 (1.91, 3.17)	2.41 (1.40, 4.15)	1.80 (0.65, 5.01)
Waist circumference, per 1 cm	-	-	0.99 (0.97, 1.01)	0.99 (0.97, 1.01)	0.99 (0.97, 1.01)	1.00 (0.95, 1.04)	0.99 (0.96, 1.02)	0.99 (0.96, 1.02)	0.99 (0.96, 1.03)
Current smoking, y/n	-	-	-	1.15 (0.69, 1.93)	1.15 (0.69, 1.93)	1.15 (0.69, 1.93)	1.33 (0.63, 2.81)	0.99 (0.53, 1.85)	1.07 (0.48, 2.39)
Physical activity, y/n	-	-	-	0.87 (0.65, 1.23)	0.87 (0.65, 1.23)	0.87 (0.65, 1.23)	0.99 (0.71, 2.14)	0.97 (0.73, 1.31)	0.89 (0.68, 1.31)
MedDietScore, per 1 unit (0–55)	-	-	-	0.96 (0.94, 1.02)	0.96 (0.94, 1.02)	0.96 (0.94, 1.02)	0.99 (0.96, 1.05)	0.97 (0.94, 1.02)	0.96 (0.94, 1.02)
Diabetes mellitus, y/n	-	-	-	-	1.81 (0.93, 3.52)	2.12 (0.84, 5.33)	1.55 (0.78, 3.07)	1.71 (0.32, 3.18)	1.44 (0.69, 3.01)
Hypertension, y/n	-	-	-	-	1.31 (0.75, 2.29)	1.34 (0.51, 3.17)	1.01 (0.53, 1.92)	1.41 (0.81, 2.47)	1.34 (0.67, 2.68)
Family history of CVD, y/n	-	-	-	-	1.55 (0.61, 1.79)	1.57 (0.72, 3.45)	1.34 (0.74, 2.42)	1.01 (0.60, 1.70)	1.19 (0.64, 2.21)
HDL-C, per 1 mg/dL	-	-	-	-	0.97 (0.95, 1.01)	1.03 (0.95, 1.05)	1.03 (0.96, 1.06)	1.02 (0.99, 1.04)	1.02 (0.99, 1.06)
TGL, per 1 mg/dL	-	-	-	-	0.99 (0.98, 1.01)	1.01 (0.99, 1.02)	1.01 (0.99, 1.02)	1.00 (0.99, 1.01)	1.01 (0.99, 1.02)
HOMA-IR, per 1 unit	-	-	-	-	-	1.12 (1.02, 1.22)	-	-	-
usCRP, per 1 mg/L	-	-	-	-	-		1.04 (1.02, 1.11)	-	-
White blood cells, per 1 count	-	-	-	-	-	-	1.20 (1.01, 1.48)	-	-
Arterial distensibility, per 1000 mmHg^−1^	-	-	-	-	-	-	-	0.90 (0.74, 0.96)	-
Total testosterone, per 1 ng/dL	-	-	-	-	-	-	-	-	0.99 (0.97, 1.01)

Hazard ratios and their corresponding confidence intervals were obtained through Cox regression analysis. Abbreviations: Hazard ratio (HR); High density lipoprotein cholesterol (HDL-C); Homeostatic Model Assessment of Insulin Resistance (HOMA-IR); Skeletal muscle mass index (SMI); Triglycerides (TGL); ultra-sensitive C-reactive protein (usCRP); White blood cells (WBC); yes/no (y/n); 95% Confidence Interval (95%CI). Bold indicates statistical significant outcomes, i.e., *p*-value < 0.05.

**Table 3:** Sensitivity analysis to evaluate the association of SMI with 4-year first fatal/non-fatal CVD event in apparently healthy men and women of IKARIA study according to participants’ age and gender (*n* = 1141).

Models	Standard Model	Standard Model Plus HOMA-IR	Standard Model Plus usCRP and WBC	Standard Model Plus Arterial Distensibility	Standard Model Plus Total Testosterone
	HR (95%CI)	HR (95%CI)	HR (95%CI)	HR (95%CI)	HR (95%CI)
**Total sample excluding participants with age <65 years**					
2nd vs. 1st SMI tertile	**0.80 (0.45, 0.96)**	0.95 (0.36, 1.31)	1.03 (0.59, 1.56)	0.92 (0.45, 1.38)	1.07 (0.52, 1.42)
3rd vs. 1st SMI tertile	1.20 (0.61, 2.35)	1.15 (0.53, 2.53)	1.29 (0.55, 3.06)	1.33 (0.53, 3.38)	1.13 (0.42, 2.71)
*p* for age interaction = 0.003					
**Men**					
2nd vs. 1st SMI tertile	0.87 (0.56, 1.85)	0.94 (0.30, 1.93)	0.87 (0.30, 2.54)	1.04 (0.43, 2.15)	0.88 (0.29, 2.62)
3rd vs. 1st SMI tertile	**0.64 (0.36, 0.99)**	**0.74 (0.41, 0.98)**	**0.58 (0.13, 0.89)**	0.84 (0.54, 1.42)	0.59 (0.25, 1.55)
**Women**					
2nd vs. 1st SMI tertile	**0.71 (0.33, 0.95)**	1.10 (0.34, 1.99)	1.12 (0.36, 3.45)	0.75 (0.19, 1.71)	**0.79 (0.40, 0.99)**
3rd vs. 1st SMI tertile	1.42 (0.50, 4.03)	1.10 (0.23, 4.31)	1.91 (0.51, 4.66)	1.52 (0.47, 4.91)	1.89 (0.46, 4.76)
*p* for gender interaction = 0.01					
**Men excluding participants with age < 65 years**					
2nd vs. 1st SMI tertile	0.92 (0.40, 1.07)	1.03 (0.22, 1.89)	1.21 (0.40, 3.71)	1.19 (0.38, 3.80)	0.99 (0.29, 1.35)
3rd vs. 1st SMI tertile	0.69 (0.51, 1.10)	0.73 (0.65, 1.19)	0.74 (0.46, 1.21)	0.70 (0.51, 1.09)	0.75 (0.42, 1.16)
**Women excluding participants with age < 65 years**					
2nd vs. 1st SMI tertile	**0.59 (0.19, 0.89)**	**0.58 (0.14, 0.86)**	0.89 (0.36, 1.50)	0.63 (0.17, 1.09)	0.53 (0.14, 1.05)
3rd vs. 1st SMI tertile	1.15 (0.45, 3.93)	1.29 (0.67, 4.01)	1.33 (0.68, 3.20)	1.36 (0.46, 3.98)	0.91 (0.30, 2.17)
*p* for gender and age interaction = 0.004					

Hazard ratios and their corresponding confidence intervals were obtained through Cox regression analysis. Standard model was adjusted for (age), (gender), smoking habits, MedDietScore, physical activity level, waist circumference, diabetes mellitus, hypertension, family history of cardiovascular disease, high density lipoprotein cholesterol, and triglycerides. Abbreviations: Hazard ratio (HR); Homeostatic Model Assessment of Insulin Resistance (HOMA-IR); Skeletal muscle mass index (SMI); ultra-sensitive C-reactive protein (usCRP); White blood cells (WBC); 95% Confidence Interval (95%CI). Bold indicates statistical significant outcomes, i.e., *p*-value < 0.05.

**Table 4:** Results from multivariate linear regression analysis regarding the association between skeletal muscle mass index (per 1 unit) and surrogate cardiovascular disease markers in the total sample and separately for men and women.

Total Sample			
	**Total**	**Men**	**Women**
*N*	1141	529	612
	Beta-Coefficient (standard error)	Beta-Coefficient (standard error)	Beta-Coefficient (standard error)
usCRP, per 1 mg/L	**−0.22 (0.13)**	**−0.27 (0.13)**	**−0.31 (0.13)**
Interleukin 6, per 1 pg/dL	+0.11 (0.21)	−0.10 (0.19)	−0.12 (0.20)
Tumor necrosis factor-alpha, per 1 pg/mL	+0.19 (1.18)	−0.17 (1.68)	−0.18 (0.91)
White blood cells, per 10^3^ counts	**−0.32 (0.09)**	−0.19 (0.11)	**−0.25 (0.12)**
HOMA-IR, per 1 unit	**−0.67 (0.21)**	−0.54 (0.71)	**−0.81 (0.24)**
Arterial distensibility, per 1000 mmHg^−1^	**−0.40 (0.89)**	**−0.61 (0.90)**	−0.33 (0.77)
Total testosterone, per 1 ng/dL	**+0.19 (0.11)**	**+0.27 (0.16)**	+0.08 (0.10)
**Total sample excluding participants <65 years old**			
	**Total**	**Men**	**Women**
*N*	670	327	343
usCRP, per 1 mg/L	**−0.21 (0.12)**	−0.15 (0.11)	**−0.38 (0.15)**
Interleukin 6, per 1 pg/dL	+0.12 (0.22)	−0.09 (0.17)	−0.13 (0.21)
Tumor necrosis factor-alpha, per 1 pg/mL	+0.20 (1.17)	−0.14 (1.60)	−0.17 (0.90)
White blood cells, per 10^3^ counts	−0.19 (0.25)	−0.18 (0.15)	**−0.26 (0.16)**
HOMA-IR, per 1 unit	−0.25 (0.24)	−0.25 (0.60)	−0.29 (0.27)
Arterial distensibility, per 1000 mmHg^−1^	**−0.51 (0.92)**	**−0.64 (0.87)**	**−0.45 (0.82)**
Total testosterone, per 1 ng/dL	**+0.23 (0.12)**	**+0.14 (0.13)**	**+0.19 (0.11)**

Beta-Coefficients and their corresponding standard error were obtained from linear regression analysis after adjusting for age, (gender), body mass index, physical activity, current smoking, MedDietScore, history of hypertension, diabetes mellitus, and hypercholesterolemia, and family history of cardiovascular disease. Abbreviations: Homeostatic Model Assessment of Insulin Resistance (HOMA-IR), ultra-sensitive C-reactive protein (usCRP). Bold indicates statistically significant outcomes, i.e., *p*-value < 0.05.
